# Neuromyelitis optica spectrum disorders associated with AQP4-positive-cancer—A case series

**DOI:** 10.3389/fneur.2022.1071519

**Published:** 2022-12-01

**Authors:** Yinghui Duan, Xin Wang, Xiaoyu Duan, Hanqing Gao, Xiaopei Ji, Xinyi Xiao, Feng Zhu, Qun Xue

**Affiliations:** Department of Neurology, First Affiliated Hospital of Soochow University, Suzhou, China

**Keywords:** neuromyelitis optic, autoimmune disease, neuro-oncology, aquaporin-4, cancer

## Abstract

Neuromyelitis optica spectrum disorders (NMOSD) are autoimmune, astrocytopathic diseases affecting the central nervous system(CNS), especially the central optic nerve and spinal cord. Aquaporin 4-immunoglobulin G (AQP4-IgG) is the dominant pathogenic antibody and can be detected in about 80% of patients with NMOSD. Although only a few cases were reported on NMOSD associated with cancer, they demonstrated the potential paraneoplastic link between cancer and NMOSD. In the present study, we report three NMOSD cases associated with cancer, which are teratoma and lung adenocarcinoma, teratoma, and transverse colon adenocarcinoma, respectively. Pathological staining of tumor sections revealed a high AQP4 expression. After tumor removal, all cases were stable and suffered no further relapses, which revealed the potential paraneoplastic mechanism between cancer and NMOSD. One of our patient's serum AQP4-IgG was transiently slightly elevated even though AQP4 was highly expressed in tumor cells, which indicates that AQP4 is not the main pathogenic antibody but might be induced by other underlying pathogenic antibody–antigen reactions.

## Introduction

Neuromyelitis optica spectrum disorders (NMOSD) are autoimmune, astrocytopathic diseases affecting the central nervous system (CNS). Aquaporin 4 (AQP4) was identified as the main target protein of NMOSD in 2005 (1), which enabled NMOSD to be an independent entity, apart from multiple sclerosis. Aquaporin 4-immunoglobulin G (AQP4-IgG) can be detected in about 80% of patients with NMOSD (2). Among patients with AQP4-IgG-seronegative, antibodies to myelin oligodendrocyte glycoprotein immunoglobulin G (MOG-IgG) account for 42% of all cases (3). Compared to AQP4-IgG-seropositive NMOSD, diagnostic criteria for AQP4-IgG-seronegative NMOSD are more stringent and require critical clinical criteria and additional neuroimaging findings (4). Although the incidence is extremely low, NMOSD were reported to be associated with different types of cancer, of which genitourinary, breast, and lung cancers are most frequently involved (5). NMOSD are considered paraneoplastic neurologic syndrome (PNS) as NMOSD meets the diagnostic criteria (6). We reported three NMOSD cases associated with cancer, which are teratoma and lung adenocarcinoma, teratoma, and transverse colon adenocarcinoma, respectively. Immunohistochemistry staining of the tumor sections all revealed an AQP4 high expression.

## Methods

This study reports three cases and was approved by the Ethics Committee of Soochow University, China. Written informed consent was obtained from all cases.

### Case 1

**Figure 1:**
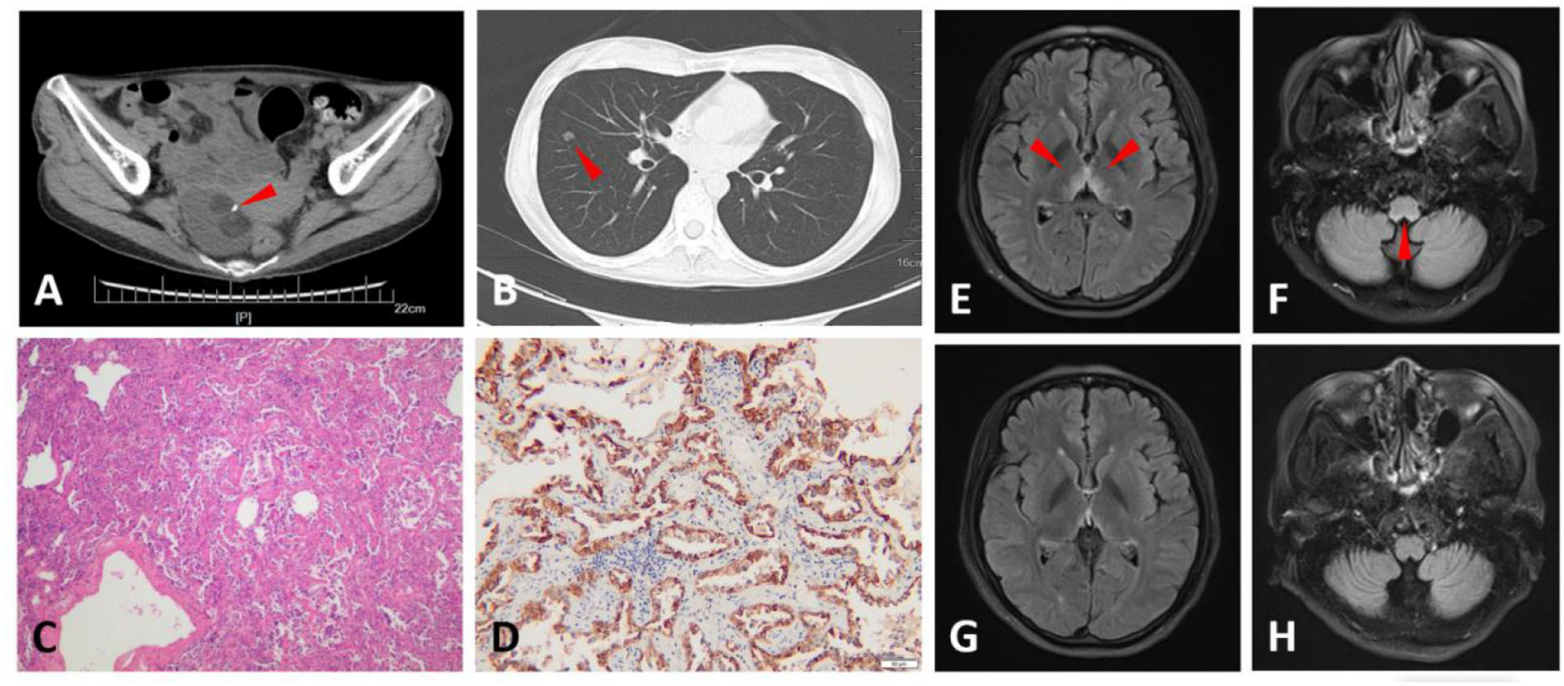
Computed tomography (CT), magnetic resonance imaging (MRI), and pathological findings of Case 1. An abdominal CT scan shows a tumor in the right ovary, in which calcification and fat were observed, and suggests an ovarian teratoma (arrowhead) **(A)**. Chest CT scan shows a nodule (9 mm in diameter) in the right lung (arrowhead) **(B)**. Hematoxylin and eosin (H&E) staining of the lung nodule section indicates adenocarcinoma **(C)**. The lung nodule section stained with aquaporin-4 (AQP-4)-specific monoclonal antibody (sc-32739, Santa Cruz, United States of America) shows intense immunoreactivity on adenocarcinoma cells **(D)**. Fluid-attenuated inversion recovery (FLAIR) images of the brain display hyperintense symmetrical lesions in the thalamus, hypothalamus **(E)**, and area postrema **(F)** when the patient was first present in our department (arrowhead). These lesions disappeared 6 months later **(G,H)**.

A 30-year-old woman presented with transient loss of consciousness, blurred vision, binaural hearing loss, tinnitus, and slurring speech. Before presenting in our department, she kept visiting the gastroenterology department and was treated there for more than 3 years because of recurrent epigastric pain, nausea, and vomiting. She underwent peroral enteroscopy and transanal enteroscopy, and no obvious abnormalities were found. The patient underwent left ovarian teratoma ablation at the age of 23 years, and she was confirmed to have teratoma in the right ovary when she was 26 years old but did not receive any treatment (Figure 1A). Her cerebrospinal fluid (CSF) demonstrated 1 leukocyte/μL, moderately elevated protein (72 mg/dL), and negativity for oligoclonal immunoglobulin G (IgG) bands (OCBs), and no neoplastic cells were found. She tested for CSF and serum AQP4-IgG, MOG-IgG, glial fibrillary acidic protein antibody (GFAP-IgG), and the autoimmune encephalitis antibody panel (N-methyl-D-aspartate receptor (NMDAR)-IgG, leucine-rich, glioma-inactivated 1 protein (LGI1)-IgG, anti-contactin-associated protein-like 2 (CASPR2)-IgG, γ-aminobutyric acid receptor (GABABR)-IgG, alpha-amino-3-hydroxy-5-methyl-4-isoxazole propionate (AMPA) receptor 1 (AMPAR1)-IgG, Alpha-amino-3-hydroxy-5-methyl-4-isoxazole propionate (AMPA) receptor 2 (AMPAR2)-IgG, IgLON Family Member 5 (IgLON5-IgG), dipeptidyl aminopeptidase-like protein 6 (DPPX)-IgG, 65-kDa glutamic acid decarboxylase (GAD65)-IgG, metabotropic glutamate receptor 5 [mGluR5)-IgG, glycine receptor (GlyR)-IgG, and anti-dopamine-2 receptor (D2R)-IgG)], which were all negative (analysis with a cell-based assay). Brain magnetic resonance imaging (MRI) showed fluid-attenuated inversion recovery (FLAIR) hyperintense and contrast-enhancing lesions in the thalamus, hypothalamus, and area postrema (Figures 1E,F). MRI was also done on the spinal cord, but no lesions were remarkable. She presented with the negativity of sero-AQP4-IgG and two core clinical characteristics (optic neuritis and area postrema syndrome); therefore, she was diagnosed with AQP4-IgG-seronegative NMOSD. She was treated with intravenous immunoglobulins (IVIG) (0.4 g/kg/d^*^5 d) and subsequent methylprednisolone (400 mg^*^3 d, 200 mg^*^3 d, 80 mg^*^3 d, 40 mg^*^3 d) and maintained with oral steroids. Six months later, her visual and hearing symptoms progressively improved, and the lesions on the cerebral MRI disappeared (Figures 1G,H). The serum AQP4-IgG was slightly elevated [3.16 U/ml; normal, <3 U/ml; ELISA (ElisaRSR AQP4 Ab Version 2, RSR Ltd, United Kingdom)] and turned negative 1 month later. Immunosuppressive treatment was planned to be initiated. However, the treatment was postponed because of the nodule in her right lung (Figure 1B). She underwent resection of the nodule in the Department of Cardiothoracic Surgery and was pathologically proven to have a lung adenocarcinoma (Figure 1C) and a high AQP-4 expression (Figure 1D).

### Case 2

**Figure 2:**
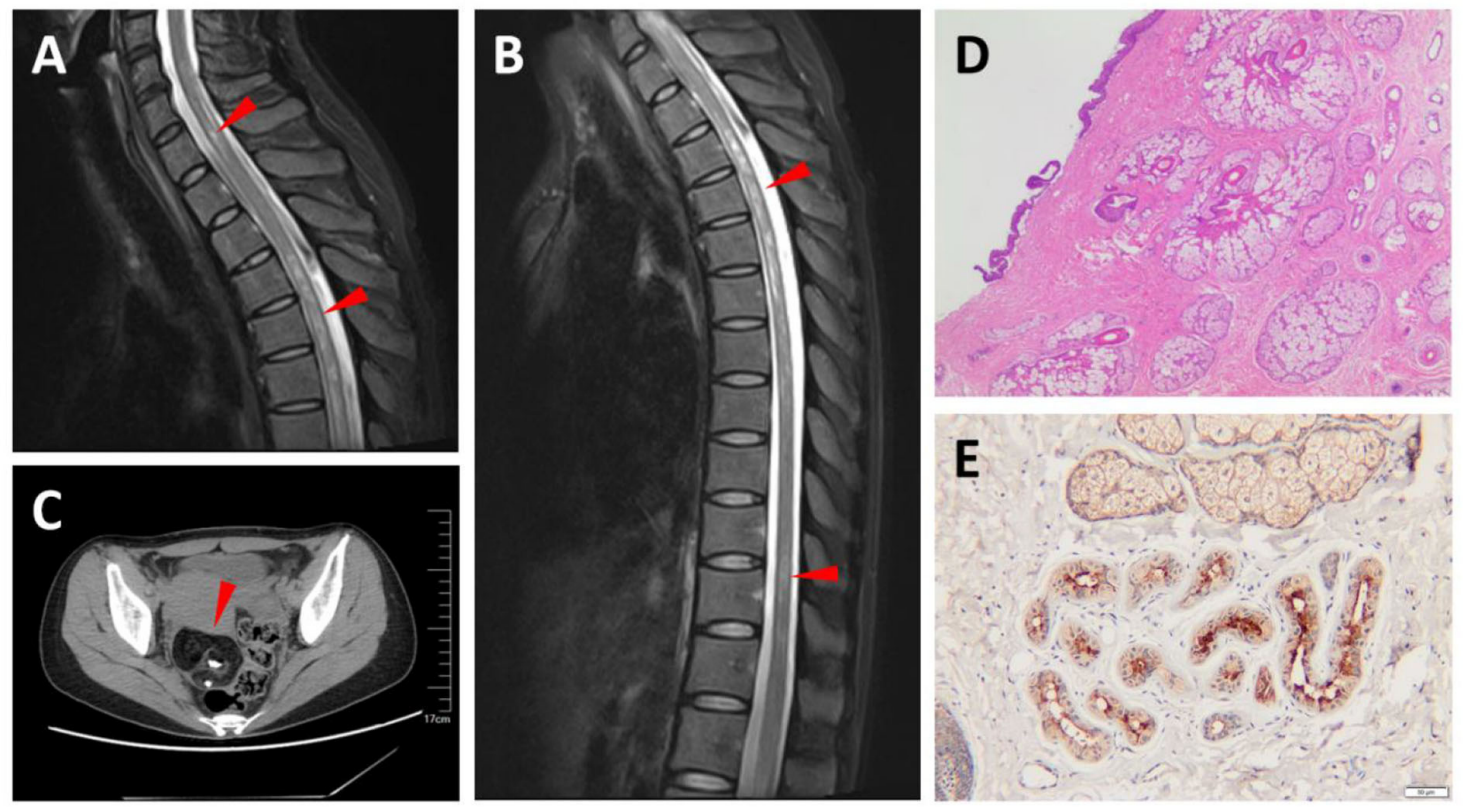
Magnetic resonance imaging (MRI) and pathological findings of Case 2. T2-weighted MRIs show abnormal signals in the cervical and thoracic spinal cord (arrowhead) **(A,B)**. An abdominal computed tomography (CT) shows a tumor in the right ovary, in which calcification and fat were observed, and suggests an ovarian teratoma (arrowhead) **(C)**. Hematoxylin and eosin (H&E) staining of teratoma section **(D)**. Teratoma section stained with aquaporin-4 (AQP-4)-specific monoclonal antibody shows intense immunoreactivity **(E)**.

This patient was a 39-year-old woman who developed numbness of lower limbs, dysuria, and defecation difficulty in 3 weeks. The cell numbers (white blood cells (WBC) 7 cells/μL) and protein (29 mg/dL) were normal in CSF and OCBs were negative. The AQP4-IgG was positive [87.94 U/ml, enzyme-linked immunosorbent assay (ELISA)] in serum while negative in CSF. She also proved positive for antinuclear antibody (ANA), anti-Sjögren's syndrome type A (SSA), and anti-Sjögren's syndrome type B (SSB) antibodies. The CSF and serum MOG-IgG and GFAP-IgG were also tested but both were negative. Spine MRI displayed T2 hyperintense segmental extensive lesions in the cervical and thoracic spinal cord (Figures 2A,B). An abdominal CT demonstrated a right ovary mass which was pathologically proved to be a mature teratoma (Figures 2C,D). Moreover, AQP-4 expression was detected in the teratoma by immunohistochemistry staining (Figure 2E). Given the positivity of sero-AQP4-IgG and transverse myelitis, she met the NMOSD diagnostic criteria. She completely recovered under intravenous methylprednisolone (500 mg^*^3 d, 250 mg^*^3 d, 120 mg^*^3 d, 80 mg^*^3 d) and was maintained with low-dose oral steroids. Serum AQP4-IgG was still positive but decreased to 21.10 U/ml 4 months later.

### Case 3

**Figure 3:**
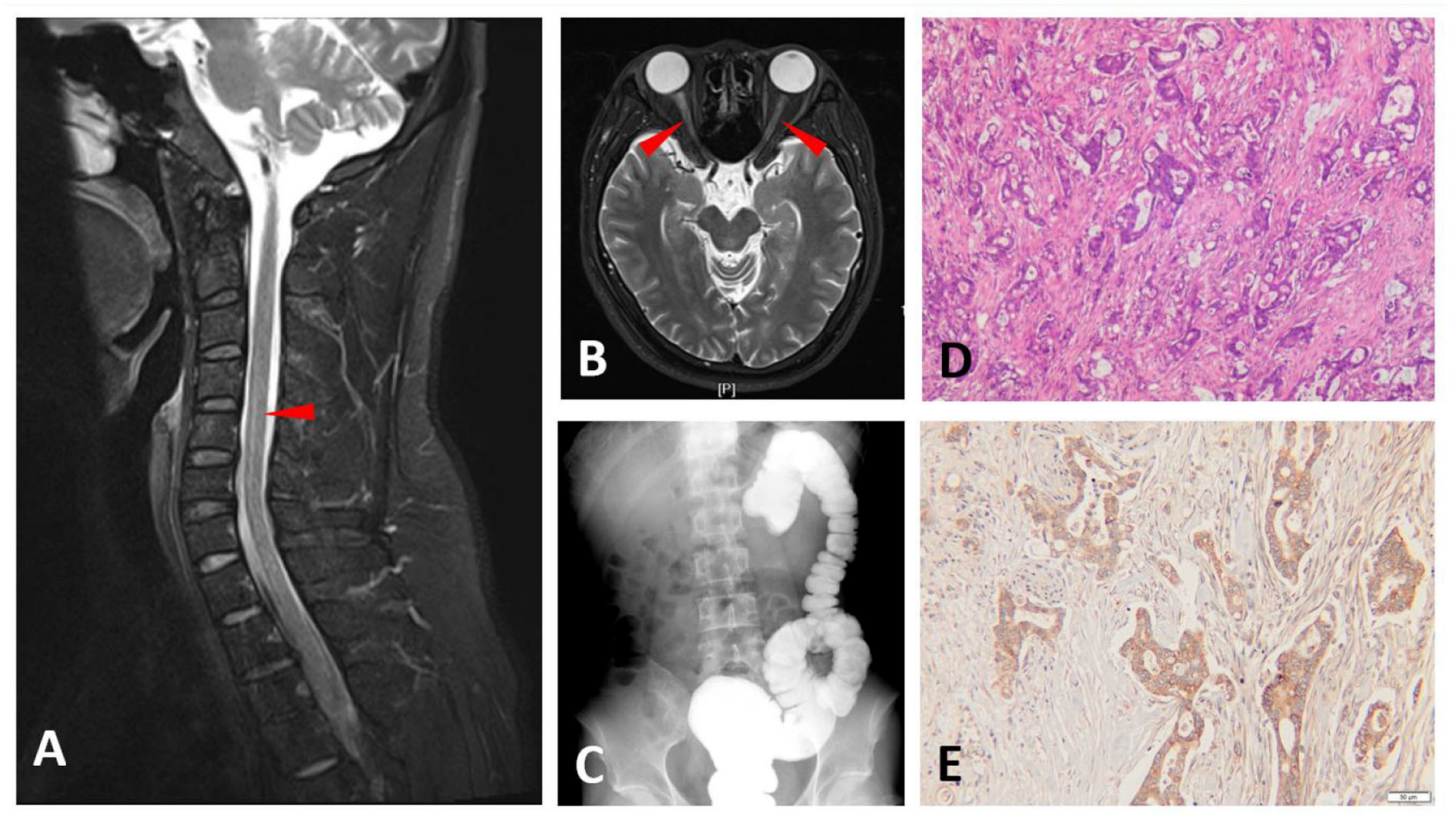
Magnetic resonance imaging (MRI), barium enema, and pathological findings of Case 3. T2-weighted MRIs show abnormal signals in the cervical spinal cord **(A)** and T2-weighted fat-suppressed MRI displayed hyperintensity in bilateral optic nerves **(B)** (arrowhead). Barium enema shows truncation of the transverse colon **(C)**. Hematoxylin and eosin (H&E) staining of the transverse colon tumor section **(D)**. Aquaporin-4 (AQP-4)-specific monoclonal antibody staining of the transverse colon tumor section shows intense immunoreactivity **(E)**.

A 25-year-old man presented with decreased visual acuity in both eyes for 2 months. Visual acuity of the right eye (VOD) was counted on fingers/before the eye, and visual acuity of the left eye (VOS) was 0.06 (best-corrected visual acuity was measured as Snellen decimal notation). The analysis of his CSF showed that the cell numbers were normal (WBC 6 cells/μL), the protein was moderately elevated (76 mg/dL), and OCBs were negative. CSF- and Sero-AQP4-IgG were both positive (5.17 and 62.44 U/ml, respectively, ELISA). MOG-IgG was positive in the serum and negative in the CSF. He was also tested for sero- and CSF-GFAP-IgG, which were both negative. The MRI showed T2 hyperintense signals in the cervical spinal cord (C4-6) (Figure 3A) and hyperintensity of bilateral optic nerves on T2-weighted fat-suppressed MRI (Figure 3B). He was diagnosed with NMOSD, as he met the NMOSD diagnostic criteria (optic neuritis and AQP4-IgG-seropositive). He received IVIG (0.4 g/kg/d^*^5d), intravenous methylprednisolone (500 mg^*^3 d, 250 mg^*^3 d, 120 mg^*^3 d, 80 mg^*^3 d), and was maintained with oral prednisolone. He was treated with rituximab two and 11 months later, respectively (600 mg/time). However, the vision of both eyes progressively declined. Three weeks after the second rituximab treatment, the visual acuity was measured. VOD was light perception and VOS was 0.04. Besides, he developed abdominal distension, nausea, and vomiting. Abdominal CT showed a mass in the transverse colon and it was histologically proved to be a tubulovillous adenoma (these tests were done in another hospital and so the images were not available). Barium enema indicated local truncation of the transverse colon (Figure 3C). Radical resection was performed which was followed by anti-tumor chemotherapy. The biopsy of the tumor was consistent with that before (Figure 3D). AQP4 staining of the adenocarcinoma was positive (Figure 3E). Two months later, the sero-AQP4-IgG decreased significantly (32.17 U/ml vs 69.28 U/ml) and his condition remained stable.

## Discussion

Paraneoplastic NMOSD cases are relatively rare, but they are considered as PNS (6), which are neurological disorders that might be triggered by antigen mimicking between tumor cells and nerve tissues followed by antibody cross-reacting. In 2021, Shahmohammadi and colleagues summarized NMOSD cases associated with cancer (5). There were 62 patients in total, of which lung adenocarcinoma (*n* = 9), gastrointestinal cancer (*n* = 7), and teratoma (*n* = 5) accounted for 14.5, 11.3, and 8.1%, respectively. After this review, two and one more paraneoplastic NMOSD cases associated with lung adenocarcinoma (7, 8) and teratoma (9) were reported respectively. Although mature teratomas are generally benign, malignant transformation occurs in 1.5%−2% of cases (10), and this makes teratoma a form of cancer.

Here, we report three NMOSD cases that were associated with AQP4-positive cancer. Case 2 and Case 3 are AQP4-IgG-seropositive, and the concentration of AQP4-IgG decreased after tumor resection and suffered no relapse. This highlights the potential paraneoplastic mechanism between cancer and NMOSD. However, Case 3 received rituximab before the operation, which could also reduce the generation of AQP4-IgG. Aquaporins (AQPs) were found to be commonly expressed in various cancer types due to the feature of trafficking water and other small molecules, which facilitate cancer development and progression. According to Dajani and colleagues, AQP4 is mainly overexpressed in brain, lung, and thyroid cancers (11). However, AQP4 was also proved to be expressed in other cancers, and the total positive rate reached 80% (5). Positive AQP4 staining of lung adenocarcinoma, gastrointestinal cancer, and teratoma was detected to be 80% (4/5), 33.3% (1/3), and 100% (5/5), respectively. AQP4 is a self-antigen and AQP4-IgG should not be generated due to immune tolerance. However, sero-AQP4-IgG-positive in these patients reveals the breakdown of self-tolerance. The possibility for this breakdown may be attributed to the fact that the structure of AQP4 on tumor cells is changed and triggers the generation of corresponding antibodies (12). AQP4 is associated with tumor growth, angiogenesis, and metastasis (11). The generation of AQP4 antibodies can prevent cancer development and spread, but meanwhile it will cause NMOSD by targeting CNS. This might be the potential mechanism of paraneoplastic NMOSD.

Ontaneda reported the first and only paraneoplastic NMOSD case associated with different types of cancer (breast carcinoma and leiomyosarcoma) to date (13). To our knowledge, Case 1 is the first paraneoplastic NMOSD case associated with both recurrent teratoma and lung adenocarcinoma. The teratomas all showed AQP4 expression in six reported NMOSD cases associated with teratoma (5, 9); therefore, we infer that the teratomas of Case 1 are AQP4 positive. However, her serum AQP4-IgG was transiently positive and at a very low concentration, even though AQP4 was highly expressed in adenocarcinoma cells. The specificity and sensitivity of the serum AQP4-assay kit used in this study are 99% and 77%. The reason may be attributed to the fact that the concentration of AQP4-IgG is too low to be detected by the present method (14). Another possibility is that AQP4-IgG is not the main pathogenic antibody but it might be induced by other underlying pathogenic antibody–antigen reactions. According to Passeri, some newly described auto-antibodies (GFAP-IgG, anti-collapsin response-mediator protein-5, anti-amphiphysin, anti-neuronal nuclear antibody-1, DPPX-IgG, GAD65-IgG, anti-Yo, anti-Ri, and others) can also mimic NMOSD (15). Unfortunately, only a few common antibodies were tested in our cases.

To conclude, herein we report three NMOSD cases associated with different types of cancer. The histological analysis demonstrates AQP4 high expression on tumor cells in all cases. They all suffered no further relapses after tumor removal. The cross-talking between NMOSD and cancer remains a mystery and is worth further in-depth research.

## Data availability statement

The original contributions presented in the study are included in the article/supplementary material, further inquiries can be directed to the corresponding author.

## Ethics statement

The studies involving human participants were reviewed and approved by Ethics Committee of Soochow University, China. The patients/participants provided their written informed consent to participate in this study. Written informed consent was obtained from the individual(s) for the publication of any potentially identifiable images or data included in this article.

## Author contributions

XW performed the immunohistochemistry staining and analyzed and interpreted the data. XD, HG, XJ, XX, and FZ collected and interpreted clinical data of patients. QX supervised the study and assisted with data interpretation and manuscript preparation. YD assembled the data and wrote the manuscript, which has been reviewed by all authors. All authors contributed to the article and approved the submitted version.

## Conflict of interest

The authors declare that the research was conducted in the absence of any commercial or financial relationships that could be construed as a potential conflict of interest.

## Publisher's note

All claims expressed in this article are solely those of the authors and do not necessarily represent those of their affiliated organizations, or those of the publisher, the editors and the reviewers. Any product that may be evaluated in this article, or claim that may be made by its manufacturer, is not guaranteed or endorsed by the publisher.
